# Tumor Necrosis Receptor Superfamily Interact with Fusion and Fission of Mitochondria of Adipose Tissue in Obese Patients without Type 2 Diabetes

**DOI:** 10.3390/biomedicines9091260

**Published:** 2021-09-18

**Authors:** Daria Shunkina (Skuratovskaia), Alexandra Komar, Maria Vulf, Hung Vu Quang, Egor Shunkin, Elena Kirienkova, Anastasiia Dakchnevich, Danil Malkov, Pavel Zatolokin, Larisa Litvinova

**Affiliations:** 1 Center for Immunology and Cellular Biotechnology, Immanuel Kant Baltic Federal University, 236001 Kaliningrad, Russia; alexandkomar@gmail.com (A.K.); mary-jean@yandex.ru (M.V.); egor.shunkin@gmail.com (E.S.); elenamed@list.ru (E.K.); a.dakhnevich@gmail.com (A.D.); d.malkov161@mail.ru (D.M.); endozapa@yandex.ru (P.Z.); larisalitvinova@yandex.ru (L.L.); 2 108 Military Central Hospital, Ha Noi 11517, Vietnam; hungrgmu@gmail.com

**Keywords:** NF-kB, non-canonical NF-κB pathway, sTNFR2, sTNFRSF8, sTNFSF13, mitochondrial dynamics, fission and fusion, TFAM

## Abstract

Interactions between receptors and ligands of the tumor necrosis factor superfamily (TNFSF) provide costimulatory signals that control the survival, proliferation, differentiation, and effector function of immune cells. All components of the TNF superfamily are associated with NF-kB functions that are not limited to cell death and may promote survival in the face of adipose tissue inflammation in obesity. Inflammation dysfunction of mitochondria is a key factor associated with insulin resistance in obesity. The aim of the study was to analyze the relationship of soluble forms of receptors and ligands of the TNF superfamily in blood plasma with mitochondrial dynamics in adipose tissue (greater omentum (GO) and subcutaneous adipose tissue (Sat)) of obese patients with and without type 2 diabetes mellitus (T2DM). Increased plasma sTNF-R1, sTNF-R2, sTNFRSF8 receptors, and ligands TNFSF12, TNFSF13, TNFSF13B are characteristic of obese patients without T2DM. The TNF-a levels in blood plasma were associated with a decrease in MFN2 gene expression in GO and IL-10 in blood plasma. The TNFSF12 levels contributed to a decrease in glucose levels, a decrease in BMI, and an increase in IL-10 levels by influencing the MFN2 gene expression in GO, which supports mitochondrial fusion.

## 1. Introduction

Interactions between ligands of the tumor necrosis factor superfamily (TNFSF) and tumor necrosis factor receptor superfamily (TNFRSF) provide co-stimulation signals that control the survival, proliferation, differentiation, and effector function of immune cells. Receptors and ligands can be classified into three groups. The first class is death receptors (DR), which contain the death domain (DD). The second class is receptors that interact with members of the TRAF family (TNFR-associated factor). The third class consists of decoy receptors (DcR) without intracellular interacting partners that act as inhibitors of the TNFSF ligand [1].

TNFR1 belongs to the first class of receptors and is expressed in various types of cells. TNFR1 is a DR and contains a death domain DD. TNFR1 is activated by cytotoxicity signaling pathways and promotes an acute inflammatory response. TNFR1 activation induces apoptosis or necroptosis and activates nuclear factor-κB (NF-κB) [1].

TNFR2 belongs to the second class of receptors, does not contain a death domain, and does not cause cell death. TNFR2 stimulates the signaling of the non-canonical NF-κB pathway. Circulating sTNFR2 has been shown to increase in response to TNF and is negatively associated with insulin sensitivity in obese patients [2].

Another receptor of this class, TNFRSF8 (CD30), forms an immune response, TNF production, and NF-κB activation [3]. TNFRSF8 can promote cell proliferation and survival or suppress replication and lead to apoptosis, depending on other signals and factors [1]. CD30 has many functions that depend on both the microenvironment and target cells.

The second class of receptors has several ligands, TNFSF12 (or tumor necrosis factor-like weak inducer of apoptosis (TWEAK)). TNFSF12 is expressed by leukocytes and is degraded to a soluble form in response to inflammatory stimuli [4]. TNFSF12 activates the NF-κB, ERK1/2, and p38 pathways and regulates proliferation, migration, differentiation, apoptosis, angiogenesis, and inflammation [4]. The role of TNFSF12 in adipose tissue (AT) and obesity is controversial. The competitive interference ability of sTNFSF12 in TNFα signaling in adipocytes was revealed—sTNFSF12 acted as a protective element in type 2 diabetes mellitus (T2DM) [4].

A proliferation-inducing ligand (APRIL/TNFSF13) and B-cell activating factor (BAFF/TNFSF13B) are associated with B-cell survival and differentiation [5]. TNFSF13 and TNFSF13B bind to two receptors expressed at different stages of B-cell development, a transmembrane activator, and calcium modulator, as well as interacting with cyclophilin ligand (TACI) and B-cell maturation protein (BCMA) [5]. TNFRSF13B at 17p12 was identified as a gene influencing the pleiotropy of metabolic syndrome [6]. According to the study, TNFRSF13B can affect cell adhesion and differentiation of adipocytes, insulin/glucose sensitivity, cytokine efficiency, plasma lipid levels, and lipoprotein density [6].

All of the above receptors and ligands of the TNF superfamily are associated with NF-kB, activated in two ways—canonical and non-canonical [7]. The canonical NF-κB pathway is activated mainly due to proinflammatory receptors and genotoxic agents [8]. The non-canonical activation of NF-κB is stimulated by family members of specific TNF receptors belonging to the second and third classes [8].

Mitochondria are key organelles that control the physiological role of adipocytes: differentiation, lipid homeostasis, insulin sensitivity, oxidative capacity, adaptive thermogenesis, and browning of white AT. Adipose tissue is an endocrine organ and a significant contributor to chronic inflammation in obesity [9]. Interestingly, subcutaneous adipose tissue (SAT) and VAT have several differences [10].

Mitochondrial function is suppressed in obesity [11]. In conditions of excess free fatty acids into the cell in obesity, mitochondria try to compensate for this, but the reserves are depleted over time [10]. In mitochondria, changes in the electron transport chain (ETC) and membrane potential were found, which led to a change in the permeability of the mitochondrial membrane and reduced the synthesis of ATP [12]. Mitochondria are highly plastic and participate in dynamic processes such as mitochondrial fusion and division. It is completely unknown why mitochondria fission and fusion, but researchers believe that mitochondrial DNA (mtDNA) is exchanged [10]. In addition, mitochondria regulate fusion and fission based on the number of nutrients to meet the cell’s needs effectively. The fission and fusion processes are referred to as mitochondrial dynamics. The balance between mitochondrial fission and fusion is the central mechanism of bioenergetic adaptation to the cell’s metabolic needs.

The process of mitochondrial fusion is regulated by the proteins GTPases, mitofusin 1 and 2 (MFN1/2), and the product of the *OPA1* gene, which are located on the outer and inner membranes mitochondria, respectively [10]. Once close contact is established between mitochondria, the fusion of the outer mitochondrial membranes occurs by forming a complex between MFN1 and MFN2. It has been reported that decreased *MFN2* expression is associated with decreased mitochondrial function in subcutaneous and visceral VT in obese patients [10]. The mitochondrial fusion process maintains genetic and biochemical homogeneity, regulating elevated levels of reactive oxygen species (ROS) and mutated DNA.

Mitochondrial dynamics are essential for maintaining a healthy mitochondrial population within the cell. It was found that high glucose content can activate FIS1, which leads to increased fragmentation of mitochondria and overproduction of ROS [12].

Thus, it is assumed that the processes of mitochondrial fusion are induced under conditions of optimization of mitochondrial bioenergetics. In contrast, the division functions are associated with the degradation of mitochondria (damage to mitochondria). Successful control of these processes can provide several therapeutic approaches, one of which is the modulation of processes associated with mitochondria: mitochondrial dynamics, apoptosis, and autophagy.

All of the above indicates the relationship between the superfamily TNF and NF-κB signaling with mitochondrial biogenesis [13,14]. A deeper understanding of the relationship between NF-κB and mitochondrial dynamics is an important area of research for regulating genes and pathways activated in chronic inflammation.

Thus, the study aimed to analyze the relationship of soluble forms of receptors and ligands of the TNF superfamily in blood plasma with mitochondrial dynamics in adipose tissue (greater omentum (GO) and subcutaneous adipose tissue (Sat)) of obese patients with and without T2DM.

## 2. Materials and Methods

### 2.1. Serum/Plasma Blood Studying

Thirty-three conditionally healthy donors (BMI = 22.5 ± 2.5 kg/m^2^, 39 ± 8 years, 11 men and 22 women), 118 obese patients without T2DM (41.8 ± 7.0 kg/m^2^, 42 ± 10 years, 30 men and 88 women) and 196 obese patients with T2DM (45.1 ± 8.7 kg/m^2^, 45 ± 9 years, 41 men and 155 women) were studied.

Samples of biological material (biopsies from SAT and GO) from obese patients were collected during different types of bariatric surgery according to the indication: Gastric bypass or longitudinal resection. During routine laparoscopic surgeries, tissue biopsies from healthy donors were taken: Inguinal and femoral hernias (right or left), diaphragmatic and abdominal hernias, and nephroptosis. Patients with type 2 diabetes take metformin at a dose of 500–1500 mg per day. Patients with type 2 diabetes did not receive insulin therapy.

Newly diagnosed cases of T2DM were found in 18% of the patients who participated in the study. The duration of T2DM in the studied patients was 2.0 ± 1.3 years. All blood samples were collected in the morning on an empty stomach according to the protocol. The value of glycated hemoglobin (HbA1C) was 5.21 (5.1–5.4) % in the control group, 5.81 (5.6–5.9) % in the group of obese patients without T2DM, 7.59 (6.45–8.67) % in the group of obese patients with T2DM. Glucose values after the test breakfast were 5.5 ± 0.8 mmol/l in the control group, 6.2 (5.8–7.1) mmol/l in obese patients without T2DM, 11.03 ± 5.23 mmol/l in the group of obese patients with T2DM. Venous blood samples were taken 60 min after the test meal for glucose determination. Breakfast consisted of Buckwheat milk porridge without sugar (200 g), jelly without sugar (150 g). The test breakfast contained 9.1 g protein, 88.1 g carbohydrate, and 10.67 g fat. The total calorie content of the test breakfast was 466 kcal.

The presence of obesity and T2DM was established on the basis of a detailed clinical and instrumental examination in a specialized hospital, guided by the World Health Organization (1999–2013) criteria for diagnosing diabetes and other types of hyperglycemia [15]. Informed consent was signed by all patients. Verification of the diagnosis and recruitment of patients into the study groups was carried out at the Department of Reconstructive and Plastic Surgery on the basis of a regional clinical hospital in Kaliningrad.

The study was conducted according to the guidelines of the Declaration of Helsinki, and approved by the Institutional Review Board (or Ethics Committee) of IKBFU (protocol code 2 and 29 November 2018).

The analysis of biochemical parameters in blood serum (glucose, cholesterol, high-density lipoproteins (HDL), low-density lipoproteins (LDL), and triglycerides) was carried out on an automatic biochemical analyzer Furuno CA-180 (Furuno Electric Company, Japan) using DiaSys test systems (DiaSys Diagnostic Systems, Holzheim, Germany).

Flow fluorimetry was used to assess the content of cytokines (sTNF-R1, sTNF-R2, sCD30/TNFRSF8, TWEAK/TNFSF12, APRIL/TNFSF13, BAFF/TNFSF13B, and IL-10) in blood plasma using the Bio-Plex Pro™ Human Inflammation Panel 1, 37-Plex #171al001m (Bio-Rad, USA) on a two-beam laser automated analyzer (BioPlex^®^ 200 Systems, Bio-Rad, USA) and BioPlexManager software (Bio-Rad, Hercules, CA, USA).

The concentration of the TNF-α molecule in the blood plasma was measured using the ELISA method (Vector-Best kits, Russia) on a Lasurit automatic enzyme-linked immunosorbent analyzer (Dynex Technologies, Chantilly, VA, USA).

### 2.2. Gene Expression Research

Total RNA from homogenized biopsies of AT was isolated using an ExtractRNA kit (Evrogen, Moscow, Russia). The resulting RNA was dissolved in 30 μL of nuclease-free water. The purity and concentration of the isolated RNA were determined using a spectrophotometer Nanovue Plus (GE Healthcare Bio-Sciences, Uppsala, Sweden). The quality of total RNA was determined by the RIN index (the RNA integrity number). The reverse transcription procedure was performed using (dT) 23 (Beagle, Moscow, Russia) and M-MLV reverse transcription (Evrogen, Moscow, Russia). To determine relative gene expression levels, qPCR was performed using qPCRmix-HS reagents (Evrogen, Moscow, Russia). As a template, we used 4 μL of cDNA, as a reference gene—*RPLPO* (a large ribosomal protein). Sequences of primers and probes for PCR are listed in Table 1.

### 2.3. Western Blot Analysis

Protein lysates were obtained using RIPA buffer (n = 8) (BioRad, Hercules, CA, USA) and normalized by the Bradford method (reagent Bradford BioRad buffer, USA) with separation by polyacrylamide gel electrophoresis (BioRad, Hercules, CA, USA). The transfer of proteins was carried out on a PVDF membrane at +4 C for 2 h (BioRad, Hercules, CA, USA). Hybridization of the target proteins was carried out with the corresponding primary antibodies and secondary antibodies labeled with horseradish peroxidase (Thermo Fisher Scientific, Waltham, MA, USA) using a substrate for visualization of Western blots ECL Plus (Thermo Fisher Scientific, Waltham, MA, USA). The analysis of the staining intensity of the bands was carried out in the Image J program with normalizations for the GAPDH protein.

Incubation with primary antibodies was performed overnight at +4 °C. Incubation with secondary antibodies conjugated with horseradish peroxidase (Thermo Fisher Scientific, Waltham, MA, USA) was carried out for 2h. Next, densitometry was measured on a ChemiDoc MP (Bio-Rad, Hercules, CA, USA) using a substrate for visualizing Western blots ECL Plus (Thermo Fisher Scientific, Waltham, MA, USA). The following primary antibodies were used: NFkB (#701079, Invitrogen, Waltham, MA, USA), mitochondrial transcription factor A (TFAM) (#MA5-16148, Thermo Fisher Scientific, Waltham, MA, USA), dynamin-1-like protein (DRP1) (#OTI3F4, Invitrogen, Waltham, MA, USA), mitofusin 2 (MFN2) (#MA5-27647, Invitrogen, Waltham, MA, USA). All target proteins were normalized the glyceraldehyde-3-phosphate dehydrogenase (GAPDH) protein (#ZG003, Thermo Fisher Scientific, Waltham, MA, USA).

### 2.4. Statistical Analysis

The normal distribution of quantitative indicators was checked using the Kolmogorov–Smirnov test and the Shapiro–Wilk test. With a normal distribution, the hypothesis of equality of the mean sample values was tested using Student’s *t*-test. The nonparametric Kruskal–Wallace test applied the significance of differences between independent quantitative samples not having a normal distribution law. In the case of statistically significant differences between the groups, analysis was performed using the Mann–Whitney test. Differences were considered significant at a significance level of *p* < 0.05.

To determine relative gene expression levels, qPCR was performed using qPCRmix-HS reagents (Evrogen, Moscow, Russia). As a template, we used 4 μL of cDNA, as a reference gene—RPLPO (a large ribosomal protein). Gene expression levels were calculated using the delta-delta Ct method, also known as 2-∆∆Ct. The formula was used to calculate the relative frequency of gene expression in the samples [16].

The analysis of the band staining intensity was carried out using the Image Lab Software (Bio-Rad, Hercules, CA, USA) with normalizations for the glyceraldehyde-3-phosphate dehydrogenase (GAPDH) protein (#ZG003, Thermo Fisher Scientific, Waltham, MA, USA).

The presence of a relationship between the studied parameters was carried out using Spearman correlation. Correlations were calculated in each group and for all patients (general correlations). For the analysis of an adequate linear regression model, the following regression residues were considered: the lack of autocorrelation of residues (Durbin–Watson test, *p*-values > 0.05) and its normal distribution and the consistency of the dispersion residues (heteroscedasticity test, *p*-values < 0.05 were considered significant). Multi-linear regression Least squares Gaussian distribution of residuals was visualized using GraphPad Prism 9.00 (GraphPad Software Inc, San Diego, CA, USA). Statistical analyzes and graphs were generated in GraphPad Prism 8.01 from the output data (GraphPad Software Inc, San Diego, CA, USA).

## 3. Results

### 3.1. Biochemical Parameters of Obese Patients

**Figure 1:**
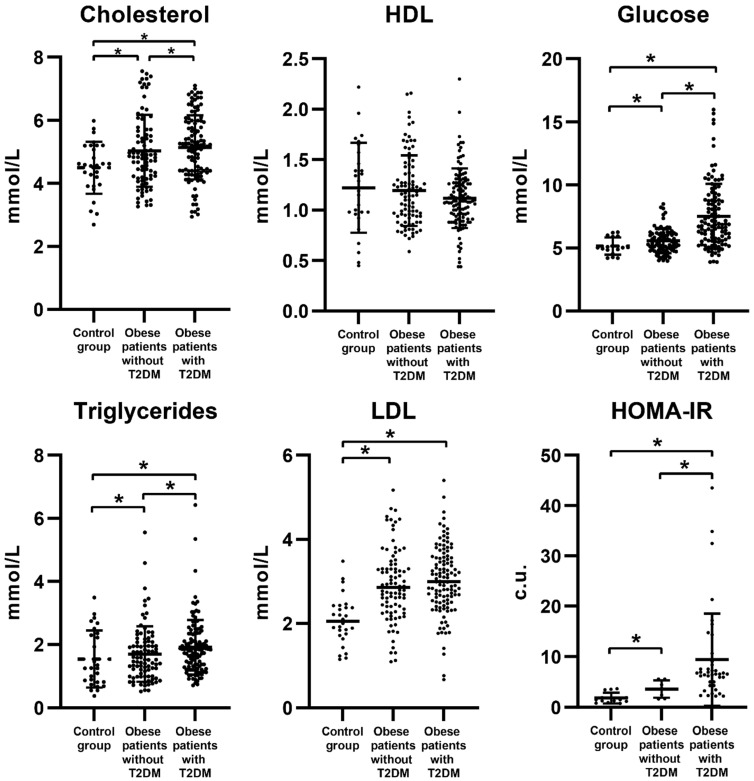
Biochemical parameters of carbohydrate and lipid metabolism in obese patients with and without T2DM. *—*p* < 0.05 the significance is determined using the Mann–Whitney criterion for two independent samples. HOMA-IR—insulin resistance index.

The results of blood biochemical parameters are presented in Figure 1. Lipid metabolism disorders were detected in obese patients with T2DM. In this category of patients, the levels of cholesterol, high-density lipoproteins (HDL), low-density lipoproteins (LDL), and triglycerides were outside the reference values. The cholesterol level in all patients was higher than in the control group (*p* < 0.05). The cholesterol in obese patients with T2DM exceeded the values of patients without T2DM (Figure 1) (*p* < 0.05). The triglycerides and LDL in serum in all obese patients were higher than in the control group. The triglycerides level reached maximum values in the group of patients with T2DM (Figure 1) (*p* < 0.05).

### 3.2. The Concentration of Plasma Cytokines of the TNF Receptor Superfamily in Obese Patients

The concentration of plasma cytokines of the TNF receptor superfamily (receptors—sTNF-R1, sTNFRSF8, and ligands—TNFSF12, TNFSF13, TNFSF13B) in obese patients with T2DM was lower than in patients without T2DM and the control group (*p* < 0.05) (Table 1). The levels of receptors sTNF-R1, sTNF-R2, sTNFRSF8, and ligands TNFSF12, TNFSF13, TNFSF13B in obese patients without T2DM were higher in comparison with the group of patients with T2DM and the control group (*p* < 0.05) (Table 2).

**Figure 2:**
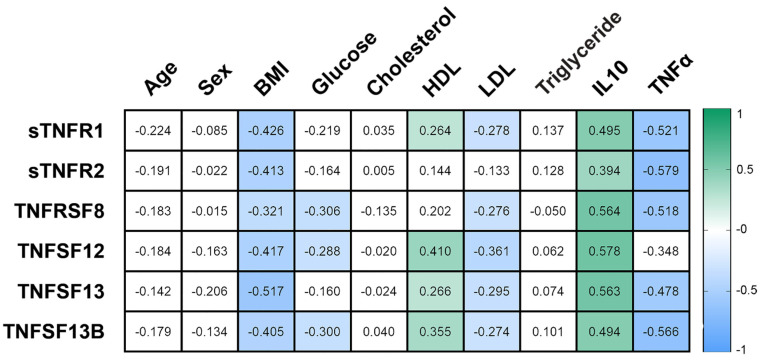
Correlation relationships of sTNF-R1, sTNF-R2, sTNFRSF8, and ligands—TNFSF12, TNFSF13, TNFSF13B with biochemical parameters in all obese patients. Filled squares show significance (*p* < 0.05).

Plasma levels of receptors and TNF ligand decreased with increasing BMI. A negative relationship was found between the levels of sTNF-R1, sTNF-R2, sTNFRSF8, TNFSF12, TNFSF13, TNFSF13B with BMI (r = −0.426; r = −0.413; r = −0.321; r = −0.417; r = −0.517; r = −0.405) (Figure 2).

The levels of sTNF-R1, sTNF-R2, sTNFRSF8, TNFSF12, TNFSF13, TNFSF13B was correlated with level of IL-10 (r = 0.495; r = 0.394; r = 0.564; r = 0.578; r = 0.563; r = 0.494; r = 0.552) general correlations (in all studied patients) (*p* < 0.05). The sTNF-R1, sTNF-R2, sTNFRSF8, TNFSF13, TNFSF13B levels was negative correlated with TNF-a levels (r = −0.521; r = −0.579; r = −0.518; r = −0.478; r = −0.566) (*p* < 0.05) general correlations (in all studied patients) (Figure 2). All studied obese patients showed a positive relationship between the levels of sTNF-R1, TNFSF12, TNFSF13, TNFSF13B with HDL levels (r = 0.264; r = 0.410; r = 0.266; r = 0.355). The levels of sTNF-R1, sTNFRSF8, TNFSF12, TNFSF13, TNFSF13B negative correlated with LDL levels (r = −0.278; r = −0.276; r = −0.361; r = −0.295; r = −0.274). Plasma levels of sTNFRSF8, TNFSF12, TNFSF13 negatively correlated with glucose levels (r = −0.306; r = −0.288; r = −0.300) in all obese patients.

**Figure 3:**
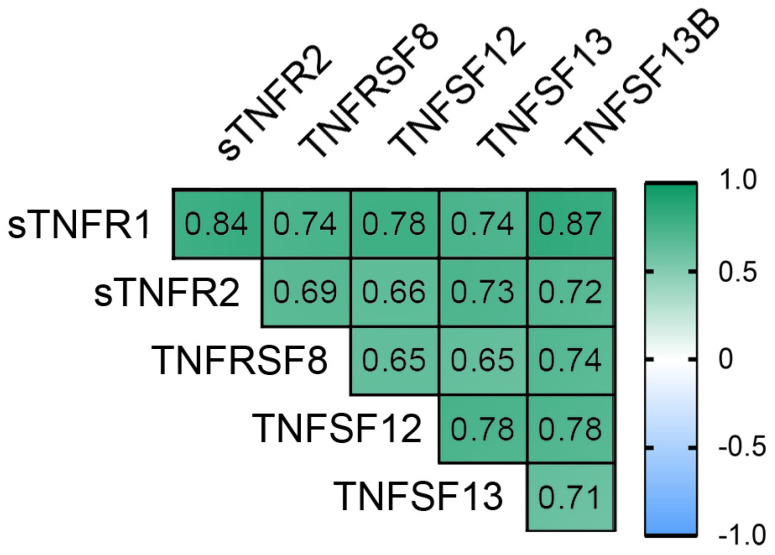
Correlation relationships between sTNF-R1, sTNFRSF8, and ligands—TNFSF12, TNFSF13, TNFSF13B. Filled squares show significance (*p* < 0.05).

sTNF-R1, sTNF-R2, sTNFRSF8, TNFSF12, TNFSF13, TNFSF13B levels were positively correlated with each other in obese patients (Figure 3).

**Figure 4:**
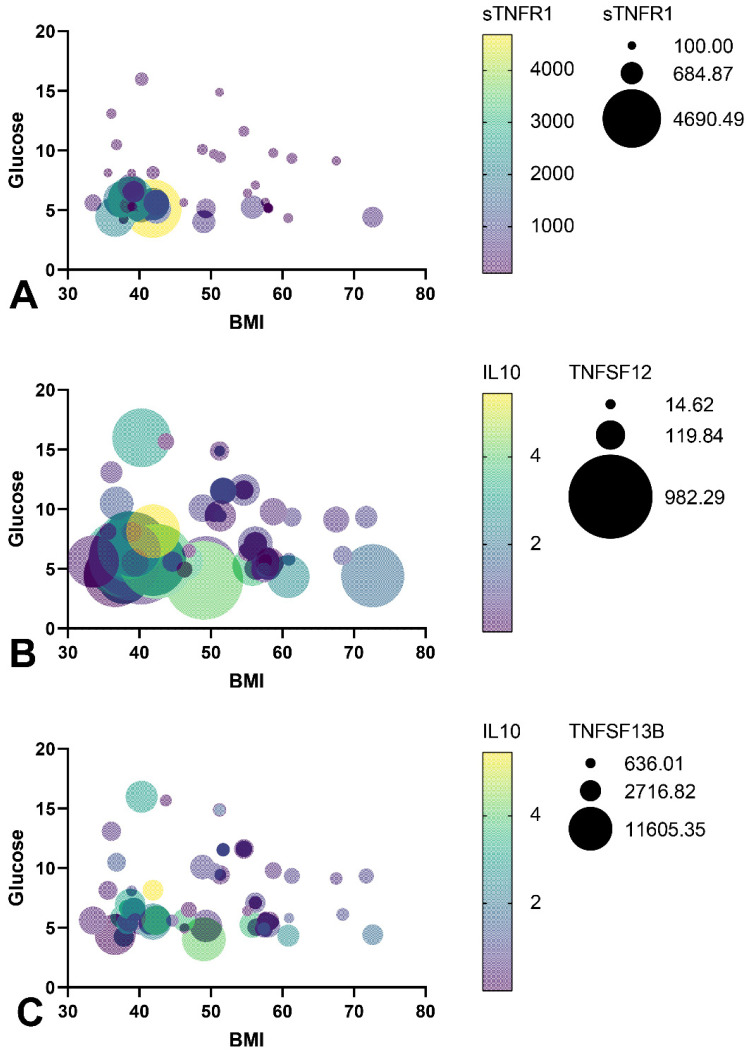
Models of multiple regression analysis of the relationship between the sTNF-R1, TNFSF12, TNFSF13B levels, and glucose level, IL10 level, and BMI (significance *p* < 0.05). (**A**) sTNF-R1 model of multiple regression; (**B**) TNFSF12 model of multiple regression; (**C**) TNFSF13B model of multiple regression.

A regression analysis was performed to prove that an increase in TNF class 2 receptors and ligands in blood plasma was not randomly associated with an improvement in carbohydrate and lipid metabolism parameters in the serum of obese patients. We analyzed whether the receptors affect the biochemical parameters of obese patients (Figure 4) (Table 3). Using multivariate regression analysis, we showed that an increase in sTNF-R1, TNFSF12, and TNFSF13B levels decreased BMI and plasma glucose levels. Multivariate regression analysis showed that an increase in TNFSF12 and TNFSF13B levels was associated with a decrease in blood glucose concentration, a reduction in BMI, and an increase in plasma IL10 levels.

Thus, increases in TNFSF12, TNFSF13B, and sTNF-R1 levels are associated with decreased glucose concentration and decreased BMI in obese patients.

### 3.3. The NF-kB Levels in Obese Patients

**Figure 5:**
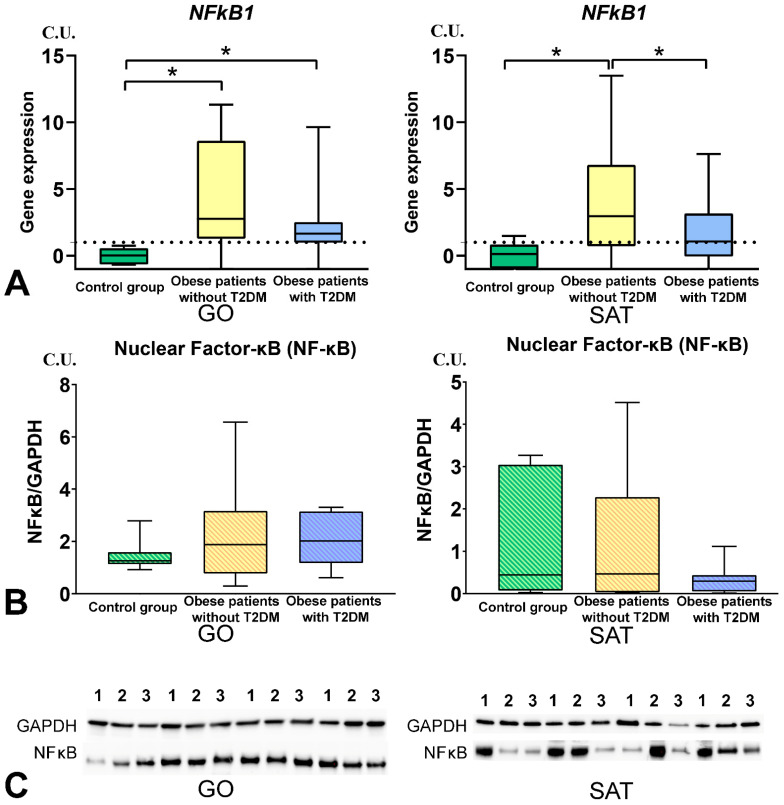
Study of NF-kB tissue-specific production in obese patients with and without T2DM. (**A**)—*NF-kB1* gene expression in GO and SAT, n = 25; (**B**)—NF-kB (p65) protein production in GO and SAT, Western blot analysis of AT biopsies, n = 8; (**C**)—Protein NF-kB (p65) Western blot bands, n = 8. *—*p* < 0.05 significance is determined using the Mann–Whitney criterion for two independent samples.

NF-kB1 gene expression in GO and SAT was increased in obese patients without T2DM compared with the control group (*p* < 0.05) (Figure 5A). NF-kB1 gene expression in GO was higher in patients with T2DM than in the control group and was not different from that of obese patients without T2DM (*p* < 0.05). NF-kB1 gene expression in SAT was lower in patients with T2DM than in obese patients without T2DM (*p* < 0.05). NF-kB (RelA) protein production in GO and SAT did not change in obese patients without T2DM compared to the control group (*p* < 0.05) (Figure 5B,C).

### 3.4. Mitochondrial Dynamics—Division and Fusion

**Figure 6:**
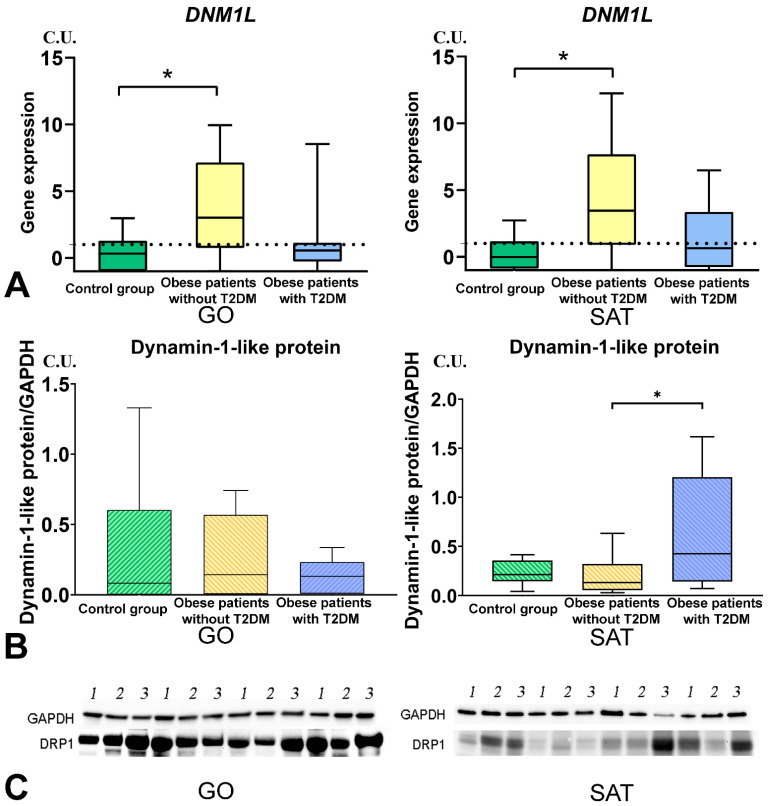
Study of tissue-specific production of DNM1L in obese patients with and without T2DM. (**A**)—*DNM1L* genes expression in GO and SAT, n = 25; (**B**)—Dynamin-1-like protein (DRP1) production in GO and SAT, Western blot analysis of AT biopsies, n = 8; (**C**)—Protein DRP1 Western blot bands, n = 8. *—*p* < 0.05 significance is determined using the Mann–Whitney criterion for two independent samples.

**Figure 7:**
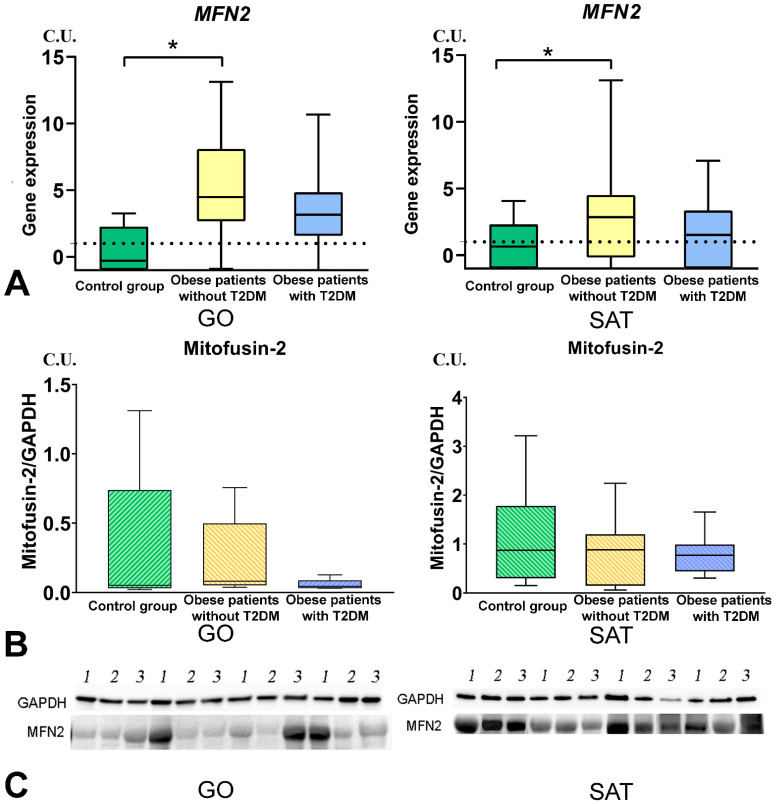
Study of tissue-specific production of MFN2 in obese patients with and without T2DM. (**A**)—MFN2 genes expression in GO and SAT, n = 25; (**B**)—Mitofusin-2 protein production in GO and SAT, Western blot analysis of AT biopsies, n = 8; (**C**)—Protein MFN2 Western blot bands, n = 8. *—*p* < 0.05 significance is determined using the Mann–Whitney criterion for two independent samples.

**Figure 8:**
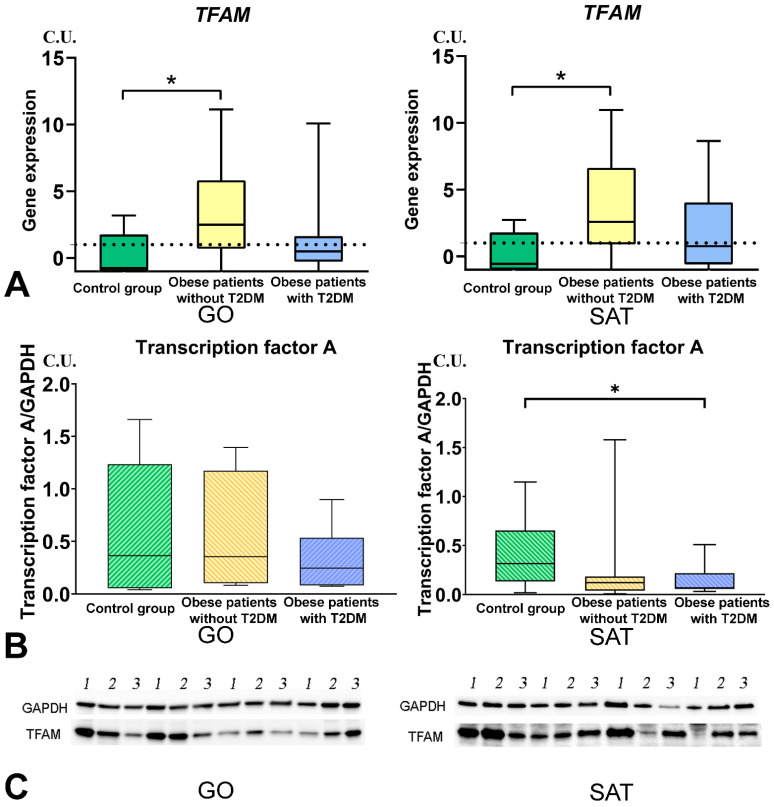
Study of tissue-specific production of TFAM in obese patients with and without T2DM. (**A**)—TFAM genes expression in GO and SAT, n = 25; (**B**)—Transcription factor A protein production in GO and SAT, Western blot analysis of AT biopsies, n = 8; (**C**)—Protein TFAM Western blot bands, n = 8. *—*p* < 0.05 significance is determined using the Mann–Whitney criterion for two independent samples.

**Figure 9:**
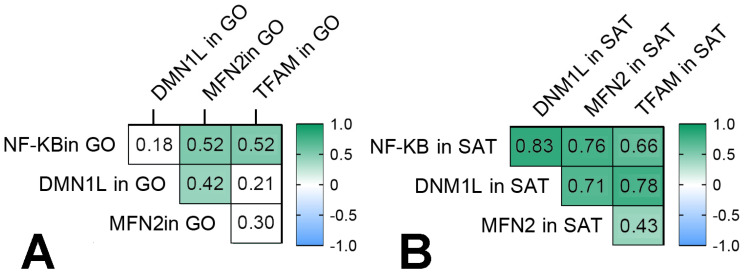
Correlation relationships of *NF-kB1, DNM1L, MFN2,* and *TFAM* gene expression in GO and SAT. Filled squares show significance (*p* < 0.05). (**A**) correlation relationships in GO; (**B**) correlation relationships in SAT.

*DNM1L* gene expression in GO and SAT was higher in obese patients without T2DM than in the control group (Figure 6). *MFN2* gene expression in GO and SAT was higher in obese patients without T2DM than in the control group (Figure 7). *TFAM* gene expression in GO and SAT was higher in obese patients without T2DM than in the control group (Figure 8). *DNM1L, MFN2*, and *TFAM* gene expression were positively correlated with each other in GO. *NF-kB1*, *DNM1L*, *MFN2*, and *TFAM* gene expression correlated positively with each other in SAT (Figure 9).

The production of dinamine-1-like protein in SAT was higher in obese patients with T2DM than in obese patients without T2DM (Figure 6). The production of transcription factor-A protein in SAT was lower in obese patients with T2DM than in the control group (Figure 5).

Thus, increased activity of mitochondrial dynamics genes is characteristic of obese patients without T2DM. In obese patients with T2DM, the production of dinamine-1-like protein was increased. In contrast, the production of transcription factor A protein was decreased compared to the other groups of patients.

### 3.5. TNF Receptors and Ligands Levels Have Been Associated with Components of Mitochondrial Dynamics

**Figure 10:**
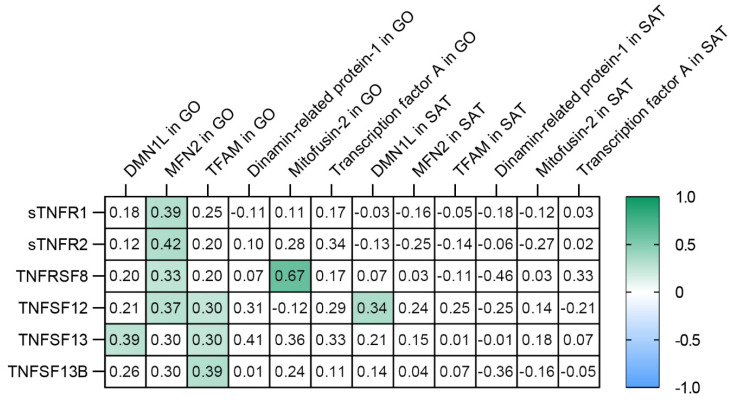
Correlations of sTNF-R1, sTNF-R2, sTNFRSF8 and ligands—TNFSF12, TNFSF13, TNFSF13B—with mitochondrial dynamics in all obese patients. Filled squares show significance (*p* < 0.05).

**Figure 11:**
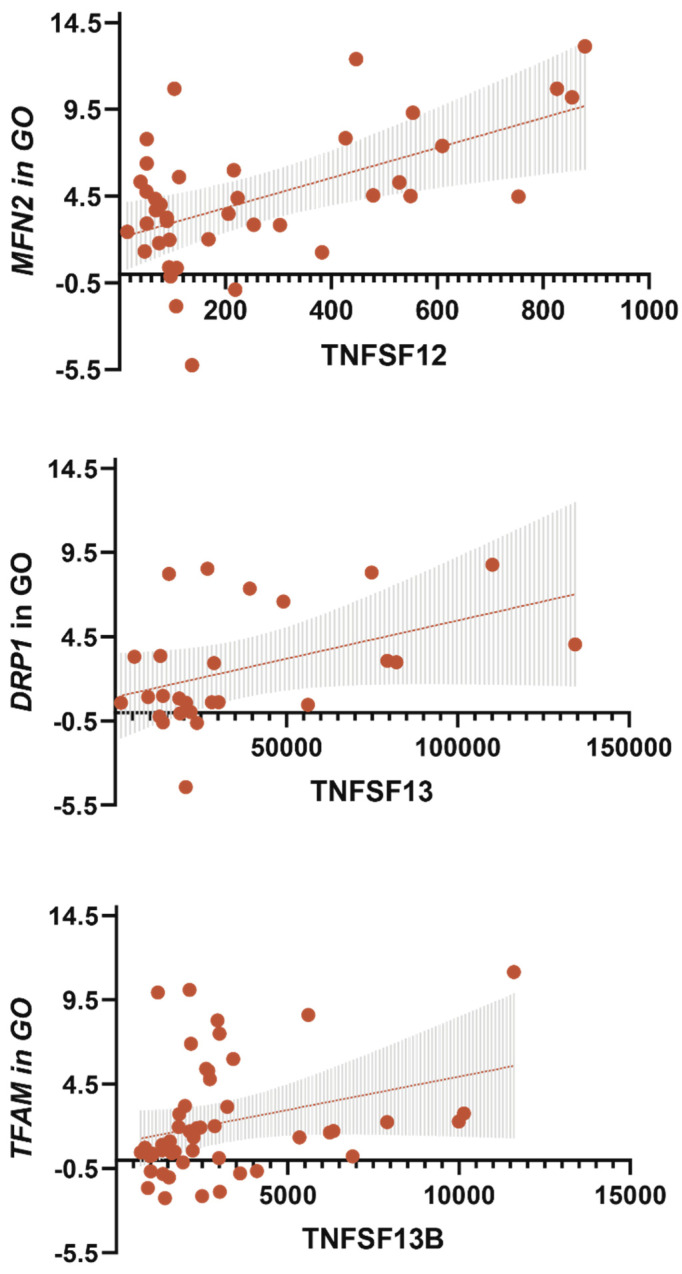
Linear regression analysis for TNF receptors and ligands with DNM1L, MFN2, and TFAM gene expression in GO.

It was found that the levels of sTNF-R1, sTNF-R2, sTNFRSF8, and TNFSF12 were positively correlated with MFN2 gene expression in GO (Figure 10 and Figure 11). The levels of TNFSF12, TNFSF13, and TNFSF13B were positively correlated with TFAM (GO) (Figure 10 and Figure 11). TNFSF13 level was positively correlated with DNM1L gene expression in GO (Figure 10 and Figure 11).

We checked if the multiple regression model included *DNM1L, MFN2,* and *TFAM* gene expression in GO and SAT. By sequential fitting, it was found that only the TNFSF12 dependent multiple regression model was statistically significant. Moreover, with the conditional parameter *MFN2* gene expression, the regression coefficient and significance increased (Table 4).

Thus, we have demonstrated an association between sTNF-R1, sTNF-R2, sTNFRSF8, TNFSF12, TNFSF13, TNFSF13B, and mitochondrial dynamics in obese patients. The most significant contribution was made by TNFSF12 level and contributed to an increase in *MFN2* gene expression in GO, an increase in IL -10 level, a decrease in glucose level, and a reduction in BMI in obese patients.

### 3.6. TNF-a Level Was Associated with MFN2 Gene Expression in GO

The multiple regression model showed that an increase in TNF-a level influenced a decrease in IL10 level and *MFN2* gene expression in GO (Table 5).

## 4. Discussion

This study investigated the role of TNF receptors and ligands in plasma of obese patients in relation to the presence or absence of T2DM. It was found that obese patients with T2DM had impaired lipid metabolism and high glucose levels. In obese patients without T2DM, levels of glucose, cholesterol, high-density lipoproteins (HDL), low-density lipoproteins (LDL), and triglycerides were predominantly within reference values (World Health Organization (1999–2013)).

Increased levels of the receptors sTNF-R1, sTNF-R2, sCD30/TNFRSF8, and the ligands TVVEAK/TNFSF12, APRIL/TNFSF13, BAFF/TNFSF13B are characteristic of obese patients without T2DM. STNFR1 and sTNFR2 can suppress the TNF-mediated inflammatory response in blood plasma [17]. This fact indicates compensatory mechanisms associated with the action of TNF receptors and ligands in obese patients without T2DM. Receptors interacting with type 2 and type 3 TNF family members may protect against metabolic complications [1]. Correlation and regression analysis of TNFSF12 and TNFSF13B levels showed that increased levels were associated with lower BMI, lower glucose levels, and increased IL10 (Figure 4 and Figure S1).

High levels of sTNF-R1, TNFSF12, TNFSF13, and TNFSF13B contribute to the maintenance of normal lipid metabolism in obese patients (*p* < 0.05). A decrease in HDL and an increase in LDL are associated with obesity. Plasma levels of sTNF-R1, TNFSF12, TNFSF13, and TNFSF13B correlated positively with HDL levels and negatively with LDL levels [18].

A positive correlation was found between the levels of sTNF-R1, sTNF-R2, sTNFRSF8, TNFSF12, TNFSF13, and TNFSF13B in blood plasma with the levels of the anti-inflammatory cytokine IL-10 and a negative correlation with TNF. IL-10, known as a factor that inhibits cytokine synthesis, is considered an essential immunoregulatory cytokine. IL-10 is compensatorily synthesized in response to the action of TNF and other proinflammatory cytokines and can inhibit inflammation under various pathophysiological conditions [19]. Data indicate the anti-inflammatory role of high concentrations of sTNF-R1, sTNF-R2, sTNFRSF8, TNFSF13, and TNFSF13B in obesity.

The levels of sTNF-R1, sTNF-R2, sTNFRSF8, TNFSF12, TNFSF13, and TNFSF13B decreased with increasing BMI in obese patients. This fact might indicate depletion of compensatory potential in obesity.

Thus, an increase in sTNFRSF8 and TNFSF13 is associated with an increase in the production of IL-10, HDL and a decrease in plasma BMI, LDL, and TNF in obese patients. Moreover, an increase in plasma levels of sTNFRSF8, TNFSF12, and TNFSF13 was associated with a decrease in serum glucose concentration in obese patients. Increased levels of sTNF-R1, sTNF-R2, sTNFRSF8, TNFSF12, TNFSF13, and TNFSF13B were found to be protective in relation to the development of T2DM in obese patients (*p* < 0.05). Multivariate regression analysis revealed that an increase in TNFSF12 and TNFSF13B levels was associated with a decrease in blood glucose concentration, a decrease in BMI, and an increase in plasma IL10 levels. Multivariate regression analysis revealed that an increase in sTNF-R1 levels was associated with a decrease in blood glucose concentration and BMI.

Adipose tissue of different localization regulates metabolism in obesity [20]. VAT is the most metabolically active fat depot [21]. Metabolic complications are thought to be associated with metabolic abnormalities in GO [20,21]. *NF-kB1* gene expression in the GO was increased in obese patients with and without T2DM compared to the control group.

SAT is considered to be a protective fat depot [22]. Anti-inflammatory cytokines and adipokines are thought to be produced in SAT [20,23]. High *NF-kB1* gene expression is unexpected in obese patients without metabolic complications. The role of NF-kB needs to be further investigated to understand why *NF-kB1* gene expression is elevated in visceral and subcutaneous adipose tissue in obese patients without T2DM, and *NF-kB1* gene expression is not as significant in patients with T2DM.

The canonical NF-κB pathway is mainly activated by proinflammatory receptors and genotoxic agents [7,8]. The canonical pathway activates the inhibitor of NF-κB kinase (IKK) complex, consisting of the IKKα and IKKβ catalytic kinases and the IKK-γ regulatory subunit (NEMO) [8]. In this case, the p105 subunit (*NFκB1* gene product) is processed to p50. P50 is associated with IκB and remains an inactive heterodimer called RelA (p65 subunit) (or c-Rel) [8]. In addition, IKK phosphorylates IκBα and ubiquitination of IκBα occurs [8]. IκBα is cleaved and releases the p50/RelA heterodimer, activating gene transcription [8].

Activation of the non-canonical pathway begins with the stabilization of NF-κB-inducing kinase (NIK). NIK phosphorizes and activates IKKα. The homodimeric IKKα complex phosphorylates and triggers the processing of p100. In addition, the p100 subunit is processed to p52, which is a distinctive feature of this pathway. Interestingly, untreated p100 inhibits DNA binding and nuclear localization of the NF-κB heterodimer [8]. p52 and its hetero- or homodimeric partner are released to bind to DNA in the nucleus and affect transcription [7,8].

The non-canonical activation of NF-κB is stimulated by family members of specific TNF receptors belonging to the second and third classes [8]. The TNFSF12, TNFSF13, and TNFSF13B levels were correlated with *NF-kB1* gene expression in GO (Figure S2). The stimulation of *NF-κB* gene expression in SAT and GO in obese patients without T2DM might be due to the activation of the non-canonical NF-κB pathway. In contrast, in obese patients with T2DM, the canonical NF-κB pathway is activated.

We found that gene expression of *NF-kB1* correlated positively with components of mitochondrial dynamics at GO and SAT (Figure 9). NF-kB can trigger genes for cell death and genes that contribute to survival under oxidative stress [8]. Recent studies have documented the importance of mitochondrial dynamics in the pathogenesis of T2DM [12,24,25,26,27]. This study shows that obese patients without T2DM have increased mitochondrial fission, fusion, and mitochondrial transcription: *DNM1L, MFN2*, and *TFAM* gene expression in GO and SAT was higher than in the control group. Judging from the change in *DNM1L* and *MFN2* gene expression, mitochondrial division and fusion are in equilibrium in obese patients without T2DM. In this study, *NF-kB1, DNM1L, MFN2*, and *TFAM* gene expression was unidirectional in GO and SAT.

The fusion of mitochondria restores their function [27,28]. Increased *MFN2* gene expression in GO and SAT may be a protective mechanism for maintaining normal mitochondrial dynamics in adipose tissue in obese patients without T2DM. We used multiple regression models and tested models for the effect of TNF receptors and ligands on *MFN2* gene expression in GO and SAT. TNFSF12 influenced the dependent variables—*MFN2* gene expression in GO, glucose level, IL10 level and BMI (r2 = 0.62, *p* < 0.05). Moreover, this multiple regression model was the strongest among all studied (Table 4). We hypothesize that TNFSF12 is involved in the maintenance of mitochondrial fusion in GO obese patients without T2DM. Plasma TNFSF12 levels contribute to the maintenance of normal carbohydrate metabolism in obese patients without T2DM.

TNF receptors and ligands in blood plasma and the expression level of mitochondrial dynamics genes in SAT and GO in obese patients with T2DM did not differ from control values. However, biochemical parameters were higher in obese patients with T2DM than in the control group. This suggests that TNF receptors and ligands and associated mitochondrial dynamics do not compensate for the development of carbohydrate and lipid metabolism complications in obese patients with T2DM.

Fission processes are associated with mitochondrial degradation and are therefore induced under conditions of mitochondrial damage [25,26]. The balance of mitochondrial dynamics is essential for metabolic health [12]. The predominance of this or that process has a pathological significance [25]. The biological value of an increase in mitochondrial fission is not completely clear. It has been reported that high glucose levels can activate mitochondrial fission 1 protein (FIS1), leading to increased mitochondrial fragmentation and overproduction of ROS [25]. The DRP1 protein production in SAT was increased in obese patients with T2DM compared to the control group. The TNFSF13 was associated with mitochondrial fission—correlation and regression analysis showed a relationship between TNFSF13 level and *DNM1L* gene expression in GO. However, TNFSF13 was not included in the multiple regression models. We hypothesize that TNFSF13 independently affects *DNM1L* gene expression, and this relationship needs further investigation.

In obese patients with T2DM, mitochondrial function is impaired under oxidative stress conditions and increased ROS production [12]. The TFAM gene plays an essential role in mitochondrial biogenesis, and cell physiology maintains mtDNA replication and transcription and regulates mtDNA copy number [28]. TFAM protein production is decreased in SAT obese patients with T2DM compared to the control group. Our previous work has provided compelling evidence for the effect of high TNF levels on high mtDNA copy numbers in adipose tissue. Moreover, in patients with morbid obesity (BMI 40 kg/m^2^), mtDNA copy number was reduced in adipose tissue compared with patients with obesity grades I and II [27,28]. Therefore, the increase in *TFAM* gene expression may indicate an increase in mtDNA transcription [25]. High *TFAM* gene expression in muscle reduces fat mass, increases energy expenditure, and decreases oxidative stress [24]. It is suggested that TFAM activation may be a therapeutic strategy for treating peripheral neuropathy [29]. In obese patients without T2DM, *TFAM* gene expression was increased in GO compared with the control group. In obese patients, correlation and regression analyzes showed a relationship between TNFSF13B and *TFAM* gene expression in GO.

The influence of TNF receptors and ligands on protection against metabolic complications in obesity, especially T2DM, is also due to blocking the effects of TNF-a. TNF-a levels correlated negatively with soluble forms of TNF receptors and ligands. Thus, a multiple regression model showed that an increase in TNF-a levels influenced a decrease in IL10 levels and *MFN2* gene expression in GO.

Thus, TNF family receptors and ligands affect the components of mitochondrial dynamics only in GO but not in SAT. TNFSF12 could promote mitochondrial fusion in GO, keep energy metabolism in balance and protect against metabolic disorders.

## 5. Conclusions

1. Increased levels of receptors sTNF-R1, sTNF-R2, TNFRSF8, and ligands TNFSF12, TNFSF13, TNFSF13B are signs of obese patients without T2DM.
2. The TNFSF12 and TNFSF13B levels were associated with NFkB1 gene expression in GO in obese patients, and *NFkB1* gene expression increased in GO and SAT in obese patients without T2DM compared with the control group. The *NFkB1* gene expression was associated with components of mitochondrial dynamic—*DNM1L, MFN2*, and *TFAM* gene expression in GO and SAT in all obese patients.
3. The *DNM1L, MFN2*, and *TFAM* gene expression levels in GO and SAT responsible for regulating mitochondrial dynamics were increased in obese patients without T2DM, and DNM1L and TFAM proteins production were unbalanced in patients with obesity and T2DM.
4. The TNFSF12 levels contributed to an increase in *MFN2* gene expression in GO; TNFSF13 contributed to an increase in *DNM1L* gene expression in GO; TNFSF13B contributed to an increase in *TFAM* gene expression in GO.
5. The TNF-a levels in blood plasma were associated with a decrease in *MFN2* gene expression in GO and IL-10 in blood plasma.
6. The TNFSF12 levels contributed to a decrease in glucose levels, a decrease in BMI, and an increase in IL-10 levels by influencing the *MFN2* gene expression in GO, which supports mitochondrial fusion.

## Figures and Tables

**Table 1:** Sequences of primers and probes for PCR.

Gene Name	Sequences of Primers and Probes
RPLPO	F: 5′-GGCGACCTGGAAGTCCAACT-3′ R: 5′-CCATCAGCACCACAGCCTTC-3′ Bgl635-5′-ATCTGCTGCATCTGCTTGGAGCCCA-3′-BHQ-2
NFkB1	F: 5′-CAGGAAGATGTGGTGGAGGA-3′ R: 5′-TGGGGTGGTCAAGAAGTAGTG-3′ FAM-5′-CCTTCTGGACCGCTTGGGTAACTCTGT-3′-BHQ-1
TFAM	F: 5′-CGCTCCCCCTTCAGTTTTGT-3′ R: 5′-TACCTGCCACTCCGCCCTAT-3′ FAM-5′-CGAGGTGGTTTTCATCTGTCTTGGCA-3′-BHQ-1
DNM1L	F: 5′-TCTGGAGGTGGTGGGGTTG-3′ R: 5′-TGGGTTTTGATTTTTCTTCTGCTAAT-3′ FAM-5′-ACCAACCACAGGCAACTGGAGAGGA-3′-BHQ-1
MFN2	F: 5′-CCAGCGTCCCATCCCTCT-3′ R: 5′-TCCACACCACTCCTCCAACA-3′ FAM-5′-ACAGGGCTCGCTCACCCAGGAG-3′-BHQ-1

**Table 2:** The level of pro- and anti-inflammatory cytokines in patients with and without T2DM.

Cytokine (pg/mL)	Control Group (n = 16)	Obese Patients without T2DM (n = 41)	Obese Patients with T2DM (n = 45)
TNF-a	2.120 (1.56–3.120)	14.71 (10.44–16.89) *p*_1–2_ < 0.001 *	32.86 (20.15–34.42) *p*_1–3_ < 0.001 * p_2–3_ < 0.001 *
sTNF-R1	110.36 (89.41–229.08)	591.57 (187.88–1229.37) *p*_1–2_ < 0.001 *	96.1 (53.05–134.83) *p*_1–3_ = 0.021 * p_2–3_ < 0.001 *
sTNF-R2	59.39 (48.03–111.73)	206.92 (107.19–288.66) *p*_1–2_ < 0.001 *	86.10 (52.21–137.23) *p*_2–3_ < 0.001 *
sTNFRSF8	31.82 (22.61–50.17)	65.13 (34.26–116.48) *p*_1–2_ < 0.001 *	23.01 (14.51–35.53) *p*_1–3_ = 0.023 * p_2–3_ < 0.001 *
TNFSF12	347.33 (247.81–464.02)	541.25 (278.36–691.23) *p*_1–2_ = 0.050*	64.15 (39.54–103.46) *p*_1–3_ < 0.001 * p_2–3_ < 0.001 *
TNFSF13	20,872.63 (17,224.93–31,761.95)	48,896.8 (28,910.78–80,400.51) *p*_1–2_ < 0.001 *	14,131.77 (8361.43–20,974.42) *p*_1–3_ = 0.001 * p_2–3_ < 0.001 *
TNFSF13B	2103.81 (1565.48–3381.11)	3411.06 (2610.11–5571.37) *p*_1–2_ < 0.001 *	1387.88 (921.86–2135.25) *p*_1–3_ = 0.001 * p_2–3_ < 0.001*
IL-10	0.64 (0.32–1.53)	2.18 (1.11–3.44) *p*_1–2_ = 0.001 *	0.75 (0.26–0.99) *p*_2–3_ < 0.001 *

Note: the significance is determined using the Mann–Whitney criterion for two independent samples (*—*p* < 0.05).

**Table 3:** Models of multiple regression analysis of the relationship between the sTNF-R1. TNFSF12, TNFSF13B levels and glucose level, IL10 level, BMI.

Multiple Regression	Dependent Variable	Independent Variable	β	Standard Error	t-Value	*p*-Value
Multiple regression linear Model 1 Multiple R-squared = 0.167, Adjusted R-squared = 0.141, *p*-value = 0.0026	sTNF-R1	BMI	−0.297	0.105	−2.819	0.0063 *
		Glucose	−0.241	0.110	−2.179	0.0329 *
Multiple regression linear Model 2 Multiple R-squared = 0.466, Adjusted R-squared = 0.438, *p*-value < 0.0001	TNFSF12	BMI	−0.325	0.082	−3.927	0.0002 *
		Glucose	−0.262	0.085	−3.082	0.0031 *
		IL10	0.439	0.122	3.585	0.0006 *
Multiple regression linear Model 3 Multiple R-squared = 0.290, Adjusted R-squared = 0.252, *p*-value = 0.0001 *	TNFSF13B	BMI	−0.251	0.093	−2.693	0.0092 *
		Glucose	−0.196	0.095	−2.052	0.0447 *
		IL10	0.341	0.137	2.479	0.0161 *

* Significance *p* < 0.05.

**Table 4:** Models of multiple regression analysis of the relationship between the sTNF-R1. TNFSF12 and TNFSF12 levels and glucose level, IL10 level, BMI, *DNM1L* gene expression in GO, *MFN2* gene expression in GO, and *TFAM* gene expression in GO.

Multiple Regression	Dependent Variable	Independent Variable	β	Standard Error	t-Value	*p*-Value
Multiple regression linear Model 4 Multiple R-squared = 0.626 Adjusted R-squared = 0.574 *p*-value < 0.0001 *	TNFSF12	BMI	−0.217	0.087	−2.493	0.0186 *
		Glucose	−0.208	0.094	−2.204	0.0356 *
		IL10	0.334	0.149	2.246	0.0324 *
		*MFN2* in GO	0.359	0.103	3.478	0.0016 *

* Significance *p* < 0.05.

**Table 5:** Multiple regression analysis models of the relationship between TNF-a level and Il-10 level and *MFN2* gene expression in GO.

Multiple Regression	Dependent Variable	Independent Variable	β	Standard Error	t-Value	*p*-Value
Multiple regression linear Model 5 Multiple R-squared = 0.702, Adjusted R-squared = 0.665, *p*-value = 6.123 × 10^−5^	TNF-a	IL10	−0.576	0.239	−2.404	0.0286 *
		MFN2 in GO	−0.526	0.112	−4.695	0.0001 *

* Significance *p* < 0.05.

## Data Availability

The data are available upon request from the author’s correspondents.

## Supplementary Materials

The following are available online at https://www.mdpi.com/article/10.3390/biomedicines9091260/s1, Figure S1: Regression Linear Analysis for TNF Receptors and Ligands, TNF Receptors and Ligands are BMI dependent in obese patients. Figure S2: Correlation relationship between all investigated parameters.

## References

[1] Sonar S., Lal G. (2015). Role of Tumor Necrosis Factor Superfamily in Neuroinflammation and Autoimmunity. Front. Immunol..

[2] Niewczas M.A., Gohda T., Skupien J., Smiles A.M., Walker W.H., Rosetti F., Cullere X., Eckfeldt J.H., Doria A., Mayadas T.N. (2012). Circulating TNF Receptors 1 and 2 Predict ESRD in Type 2 Diabetes. J. Am. Soc. Nephrol..

[3] Rinaldi I. (2018). The role of Reed-Sternberg CD30 receptor and lymphocytes in pathogenesis of disease and its implication for treatment. Acta Medica Indones..

[4] Acharya A.B., Chandrashekar A., Acharya S., Shettar L., Thakur S. (2019). Serum STWEAK Levels in Chronic Periodontitis and Type 2 Diabetes Mellitus. Diabetes Metab. Syndr..

[5] Croft M., Duan W., Choi H., Eun S.-Y., Madireddi S., Mehta A. (2012). TNF Superfamily in Inflammatory Disease: Translating Basic Insights. Trends Immunol..

[6] Zhang Y., Kent J.W., Olivier M., Ali O., Broeckel U., Abdou R.M., Dyer T.D., Comuzzie A., Curran J.E., Carless M.A. (2013). QTL-Based Association Analyses Reveal Novel Genes Influencing Pleiotropy of Metabolic Syndrome (MetS). Obesity.

[7] Suryavanshi S.V., Kulkarni Y.A. (2017). NF-Κβ: A potential target in the management of vascular complications of diabetes. Front. Pharmacol..

[8] Morgan M.J., Liu Z. (2011). Crosstalk of reactive oxygen species and NF-ΚB signaling. Cell Res..

[9] Guzik T.J., Skiba D.S., Touyz R.M., Harrison D.G. (2017). The role of infiltrating immune cells in dysfunctional adipose tissue. Cardiovasc. Res..

[10] Lee J.H., Park A., Oh K.-J., Lee S.C., Kim W.K., Bae K.-H. (2019). The role of adipose tissue mitochondria: Regulation of mitochondrial function for the treatment of metabolic diseases. Int. J. Mol. Sci..

[11] Heinonen S., Buzkova J., Muniandy M., Kaksonen R., Ollikainen M., Ismail K., Hakkarainen A., Lundbom J., Lundbom N., Vuolteenaho K. (2015). Impaired mitochondrial biogenesis in adipose tissue in acquired obesity. Diabetes.

[12] Sivitz W.I., Yorek M.A. (2010). Mitochondrial dysfunction in diabetes: From molecular mechanisms to functional significance and therapeutic opportunities. Antioxid. Redox Signal..

[13] Shi J.-H., Sun S.-C. (2018). Tumor necrosis factor receptor-associated factor regulation of nuclear factor ΚB and mitogen-activated protein kinase pathways. Front. Immunol..

[14] Hayden M.S., Ghosh S. (2014). Regulation of NF-ΚB by TNF family cytokines. Semin. Immunol..

[15] Bassols J., Moreno J.M., Ortega F., Ricart W., Fernandez-Real J.M. (2010). Characterization of herpes virus entry mediator as a factor linked to obesity. Obesity.

[16] Livak K.J., Schmittgen T.D. (2001). Analysis of relative gene expression data using real-time quantitative PCR and the 2(-Delta Delta C(T)) Method. Methods.

[17] So T., Ishii N. (2019). The TNF-TNFR family of co-signal molecules. Adv. Exp. Med. Biol..

[18] Bora K., Pathak M.S., Borah P., Das D. (2017). Association of decreased High-Density Lipoprotein Cholesterol (HDL-C) with obesity and risk estimates for decreased HDL-C attributable to obesity. J. Prim. Care Community Health.

[19] Mühl H. (2013). Pro-inflammatory signaling by IL-10 and IL-22: Bad habit stirred up by interferons?. Front. Immunol..

[20] Wronska A., Kmiec Z. (2012). Structural and biochemical characteristics of various white adipose tissue depots. Acta Physiol..

[21] Ndisang J.F., Vannacci A., Rastogi S. (2017). Insulin Resistance, Type 1 and Type 2 Diabetes, and Related Complications 2017. J. Diabetes Res..

[22] Tiller G., Laumen H., Fischer-Posovszky P., Finck A., Skurk T., Keuper M., Brinkmann U., Wabitsch M., Link D., Hauner H. (2011). LIGHT (TNFSF14) inhibits adipose differentiation without affecting adipocyte metabolism. Int. J. Obes..

[23] Yu T., Robotham J.L., Yoon Y. (2006). Increased Production of Reactive Oxygen Species in Hyperglycemic Conditions Requires Dynamic Change of Mitochondrial Morphology. Proc. Natl. Acad. Sci. USA.

[24] Koh J.-H., Johnson M.L., Dasari S., LeBrasseur N.K., Vuckovic I., Henderson G.C., Cooper S.A., Manjunatha S., Ruegsegger G.N., Shulman G.I. (2019). TFAM enhances fat oxidation and attenuates high-fat diet–induced insulin resistance in skeletal muscle. Diabetes.

[25] El-Hattab A.W., Craigen W.J., Scaglia F. (2017). Mitochondrial DNA Maintenance Defects. Biochim. Biophys. Acta Mol. Basis Dis..

[26] Luan G., Li G., Ma X., Jin Y., Hu N., Li J., Wang Z., Wang H. (2019). Dexamethasone-induced mitochondrial dysfunction and insulin resistance-study in 3T3-L1 adipocytes and mitochondria isolated from mouse liver. Molecules.

[27] Litvinova L., Zatolokin P., Vulf M., Mazunin I., Skuratovskaia D. (2019). The Relationship between the MtDNA Copy Number in Insulin-Dependent Tissues and Markers of Endothelial Dysfunction and Inflammation in Obese Patients. BMC Med. Genom..

[28] Skuratovskaia D., Zatolokin P., Vulf M., Mazunin I., Litvinova L. (2019). Interrelation of chemerin and TNF-α with MtDNA copy number in adipose tissues and blood cells in obese patients with and without type 2 diabetes. BMC Med. Genom..

[29] Chandrasekaran K., Anjaneyulu M., Inoue T., Choi J., Sagi A.R., Chen C., Ide T., Russell J.W. (2015). Mitochondrial Transcription Factor A Regulation of Mitochondrial Degeneration in Experimental Diabetic Neuropathy. Am. J. Physiol. Endocrinol. Metab..

