# Supplementary figures and images for: Tumor Necrosis Receptor Superfamily Interact with Fusion and Fission of Mitochondria of Adipose Tissue in Obese Patients without Type 2 Diabetes
Source: Biomedicines. 2021 Sep 18;9(9):1260. doi: 10.3390/biomedicines9091260 (PMC8470627; doi:10.3390/biomedicines9091260)

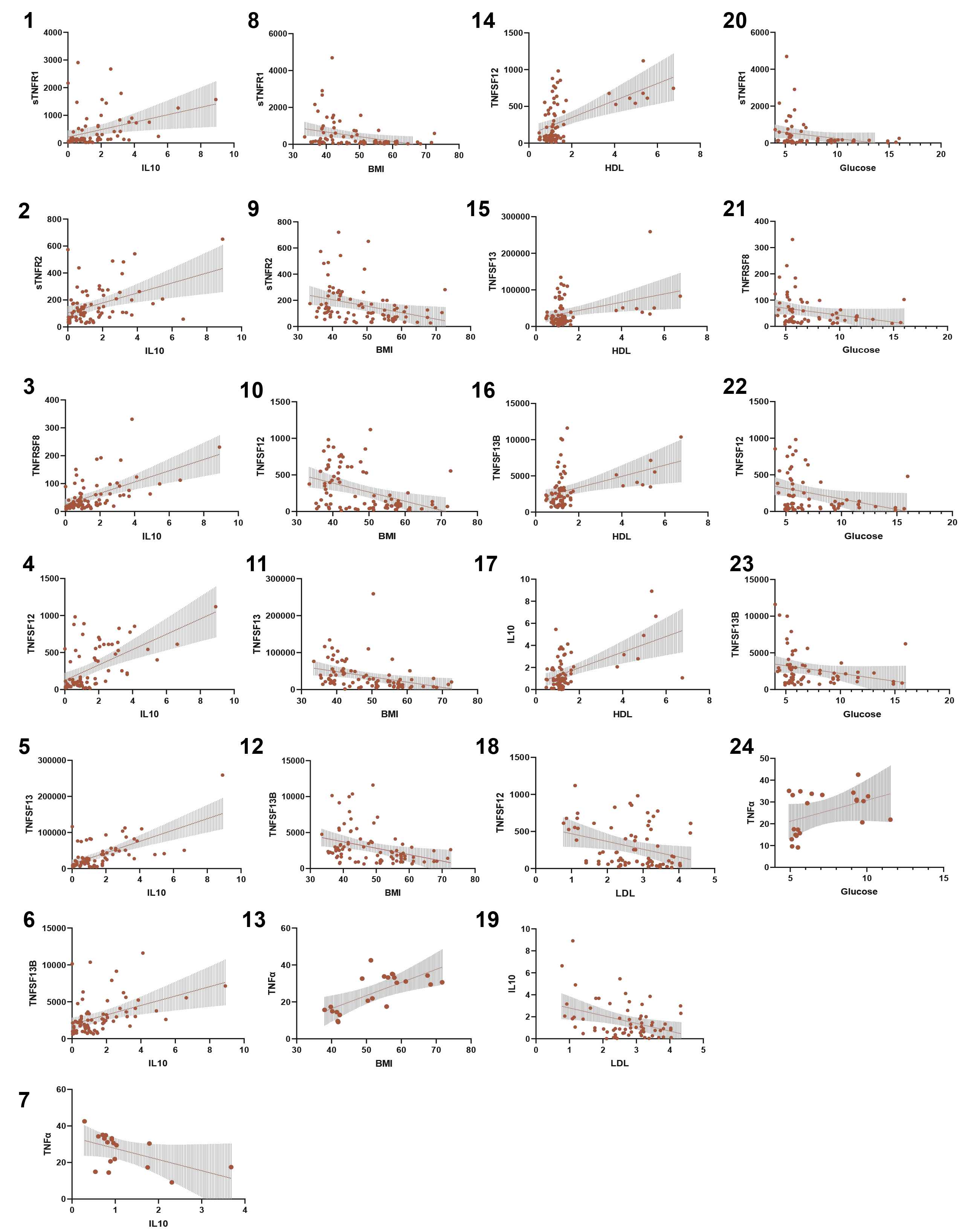

Supplement: Supplementary file 1 [file biomedicines-09-01260-s001.zip › Suppl 1.tif]

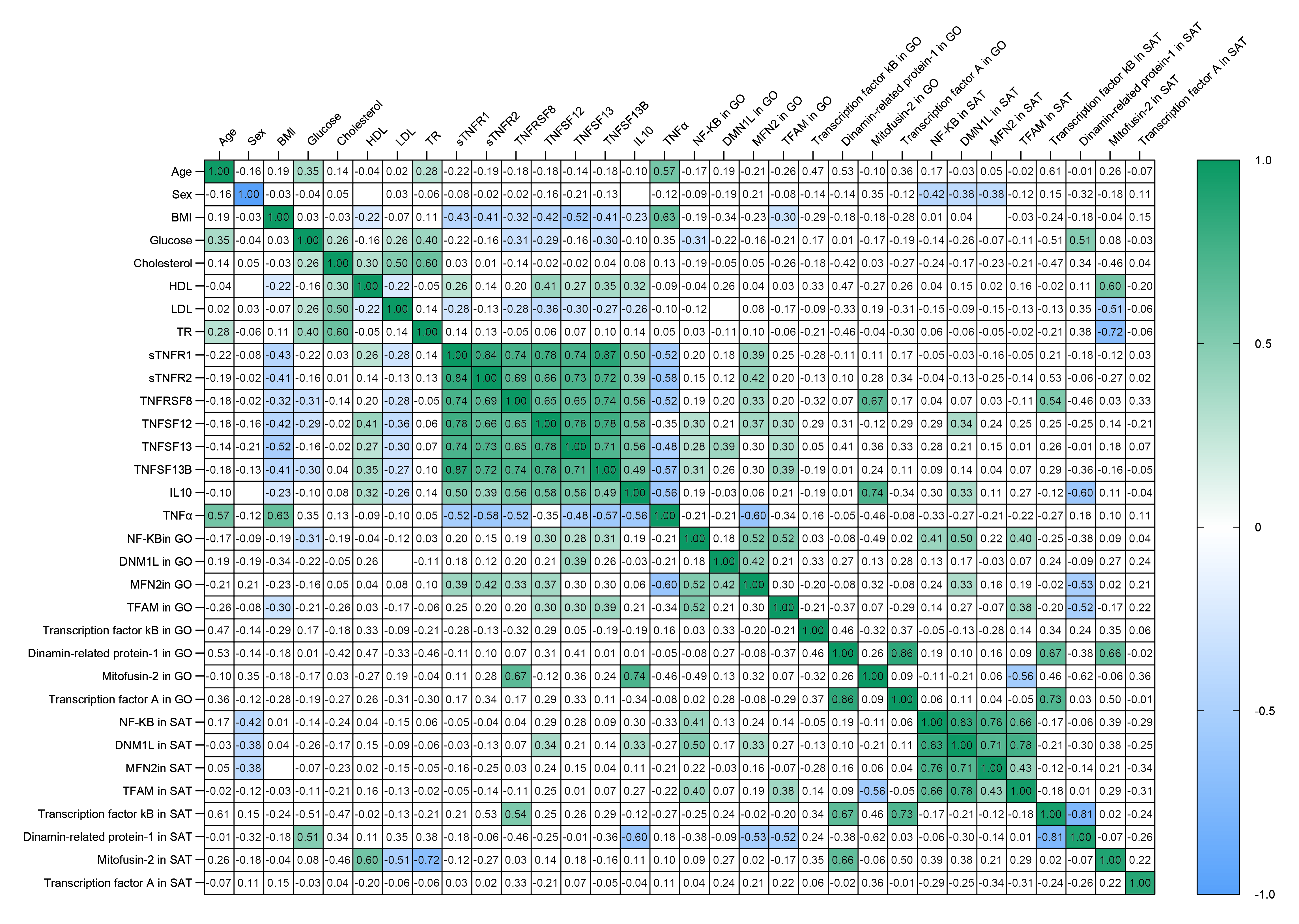

Supplement: Supplementary file 1 [file biomedicines-09-01260-s001.zip › Suppl 2.tif]
